# Histopathologie d´un rare cas de schistosomiase intramédullaire et revue de la littérature

**DOI:** 10.11604/pamj.2020.37.153.24890

**Published:** 2020-10-13

**Authors:** Christiane Judith Rissia - Ngo Pambe, David Ngaroua, Jérôme Mbo Amvene, Angèle Clarisse Kabeyene, Joseph Marie Mendimi Nkodo

**Affiliations:** 1 Faculté de Médecine et des Sciences Biomédicales de Garoua, Université de Ngaoundéré, Ngaoundéré, Cameroun,; 2 Faculté de Médecine et des Sciences Biomédicales de l´Université de Yaoundé I, Yaoundé, Cameroun

**Keywords:** Bilharziose, parasitaire, médullaire, histopathologie, Bilharziosis, parasitic, medullary, histopathology

## Abstract

La schistosomiase reste encore un problème majeur de santé publique en Afrique subsaharienne en général et au Cameroun en particulier. C´est la deuxième endémie parasitaire après le paludisme. Elle est favorisée par la coexistence de zones bioclimatiques. Nous rapportons le cas d'une fillette de 6 ans. Elle présentait un syndrome déficitaire clinique et une infiltration médullaire simulant une tumeur à l´imagerie médicale. L´intervention chirurgicale réalisée avait permis de préciser le diagnostic après l´examen histopathologique des prices biopsiques. La patiente avait en plus reçu une dose de Praziquantel. La régression des symptômes, tout comme une évolution favorable de la plaie opératoire avaient favorisé la sortie de l´hôpital. La patiente est perdue de vue depuis trois ans. La prise en charge efficace des bilharzioses neuro-méningées devrait être pluridisciplinaire.

## Abstract (en)

Schistosomiasis is a major public health problem in sub-Saharan Africa and, in particular, in Cameroon. It is the second parasitic disease endemic after malaria and it is favoured by the coexistence of bioclimatic zones. We report the case of a 6-years old girl presenting with clinical deficit syndrome and medullary infiltration mimicking tumor on medical imaging. Surgery helped to clarify the diagnosis after histopathological examination of the biopsic specimens. The patient had also received a dose of Praziquantel. Regression of symptoms as well as favorable progression of the operative wound facilitated discharge from hospital. The patient was lost to follow-up for three years. Effective management of neuromeningeal bilharziosis should be multidisciplinary.

## Introduction

Parasitose chronique due aux trématodes du genre *Schistosoma*, les schistosomiases sont un problème majeur de santé publique préoccupant, actuellement au premier plan l´OMS. Deuxième endémie parasitaire mondiale après le paludisme. Plus de 200 millions de sujets hébergent des bilharzies et 120 millions d´entre eux en sont malades (800 000 décès / an). On estime qu'au moins 92% des personnes ayant besoin d'un traitement vivent en Afrique. La schistosomiase touche plus particulièrement les populations pauvres d´agriculteurs et pêcheurs; le manque d´hygiène et certaines habitudes de jeu des enfants d´âge scolaire, telles que la natation ou la pêche dans des eaux infestées, rendent ces enfants particulièrement vulnérables à l´infection [1]. Au Cameroun, le développement des parasitoses humaines parmi lesquelles les bilharzioses urinaires et intestinales, est favorisé par l'existence d'une série de zones bioclimatiques allant de la forêt équatoriale à la savane sahélienne [2]. Les plus grands foyers de schistosomiase urinaire se trouvant dans les régions de la partie septentrionale du pays [3]. L'atteinte de la moelle épinière est rare d´où l´intérêt du cas d'une fillette de 6 ans que nous rapportons assorti d´une revue de la littérature.

## Patient et observation

Il s´agissait d´une fillette âgée de 6 ans, originaire et résidente dans la région du Nord Cameroun. Evacuée dans la capitale Yaoundé, située à plus de 1300 kilomètres du lieu de résidence habituelle pour meilleure prise en charge d´un déficit moteur d´installation brutale. Elle arrive dans le service de Neurochirurgie accompagnée de ses parents, tous aussi dépaysés qu´elle-même. Les paramètres physiques et hémodynamiques sont sans particularité. En occurrence, la patiente ne présente pas de fièvre et l´examen physique révèle un syndrome du cône terminal avec paraplégie flasque des membres inférieurs. Le tout associé à une abolition des réflexes ostéo-tendineux avec anesthésie des dermatomes L2 à S5. Le liquide céphalorachidien (LCR) montrait une pléiocytose avec hyperprotéinorachie. Les autres tests sérologiques tant sanguins que du LCR étaient non spécifiques.

**Figure 1:**
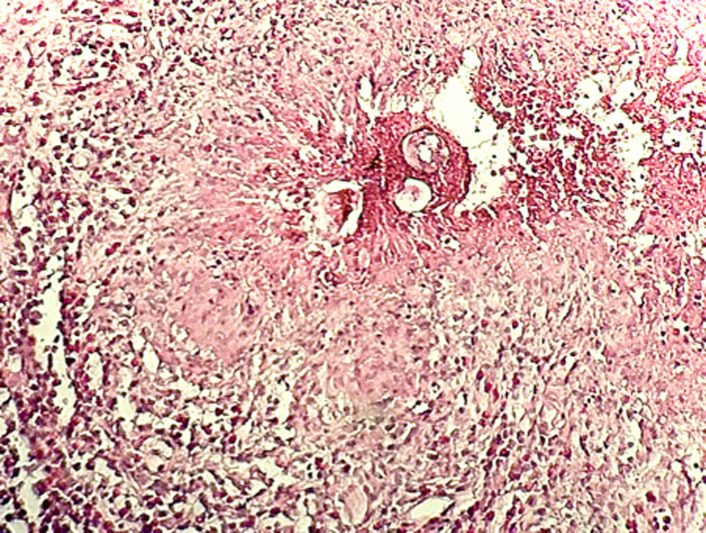
granulome inflammatoire autour d´un foyer de nécrose hébergeant des œufs de schistosome: coloration hématéine-éosine; GX100

L'imagerie par résonance magnétique (IRM) médullaire montrait une grosse moelle en regard des vertèbres dorsales D11 et 12 avec un léger hypersignal. Le diagnostic évoqué était celui d'une tumeur inflammatoire infiltrante nécessitant une intervention chirurgicale. Au cours de cette dernière, les biopsies de moelle épinière effectuées étaient transmises au laboratoire d´anatomie pathologique fixées au formol tamponné dilué au 10^e^. Macroscopiquement, ces fragments tissulaires avaient une couleur blanchâtre et une consistance friable. Ces prélèvements étaient examinés au microscope optique à fond clair après technique histopathologique standard et coloration à l'hématoxyline-éosine. L´examen microscopique au faible grandissement révélait un tissu nerveux dont l´architecture normale était modifiée et remplacée par des phénomènes inflammatoires chroniques. Ceux-ci étaient caractérisés par la présence d´un infiltrat composé de lymphocytes, plasmocytes et macrophages réalisant des foyers granulomateux. Ces derniers bordaient des zones de nécrose (Figure 1).

**Figure 2:**
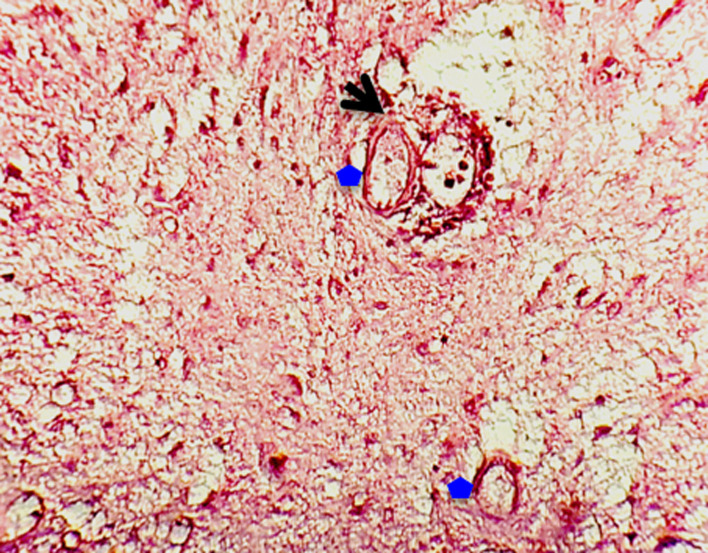
œufs de schistosome (étoiles bleues) avec éperon terminal (flèche) réalisant un bilharziome intratissulaire: coloration hématéine-éosine; GX100

A ces éléments inflammatoires, s´ajoutait un important contingent de polynucléaires éosinophiles, s'agglutinant autour et/ou recouvrant ou non par endroits des structures ovoïdes allongées, à la coque éosinophile relativement épaisse et présentant un éperon terminal (Figure 2). Le diagnostic de schistosomiase médullaire avait été retenu. L´évolution était marquée par des suites opératoires simples au 8^e^ jour. En plus des médicaments non spécifiques, la jeune patiente avait reçu un traitement à base de Praziquantel en dose unique, à raison de 40mg/kg de poids corporel. Le suivi préconisé après l'hospitalisation et l'IRM de contrôle n'avaient pas été réalisés, la patiente étant perdue de vue, aidée en cela par le fait d´être sous le contrôle des parents qui avaient décidé de rentrer dans leur localité de résidence habituelle.

## Discussion

Causée par des trématodes hématophages, la bilharziose est une affection parasitaire endémique [4]. Elle occupe le second rang après le paludisme en Afrique subsaharienne, mais on la rencontre de plus en plus en Europe occidentale, chez des sujets originaires des pays d´endémie et chez les Européens y ayant vécu. Mais le climat européen s´oppose au développement de ces affections. Si six espèces, à savoir *S. haematobium, S. mansoni, S. intercalatum, S. guinéensis, S. japonicum* et *S. mekongi* sont pathogènes pour l´homme et sévissent en Afrique, en Asie de l´Est et en Amérique du Sud; quatre d´entre elles sont retrouvées au Cameroun [3]. C´est ainsi que les personnes porteuses pourront présenter une bilharziose vésicale ou urinaire, intestinale, hépatosplénique et rectale dues respectivement à *S. haematobium, S. mansoni, S. guinéensis* et *S. intercalatum*. Situé en zone intertropicale du globe (35° de latitude Nord, 25° de latitude Sud), le Cameroun qui est donc sous l´influence des zones bioclimatiques offre un terrain favorable à l´expression de la bilharziose [2]. La contamination se fait par les femelles adultes tapies dans les fines veinules de la vessie ou de l´intestin de l´homme [5]. Celles-ci pondent des œufs qui s´éliminent avec les urines et les matières fécales. Mais ces œufs ne donneront naissance à une larve, appelée miracidium, que dans une eau douce de 20 à 25°C: marigots, rizières, ruisseaux, rivières; une hygiène rigoureuse préserverait donc de la contamination. Encore le miracidium ne vivra-t-il pas s´il ne rencontre rapidement un tout petit mollusque d´eau douce, nécessaire à son développement et spécifique pour chaque espèce: il s´agit d´un bullin pour *S. haematobium*, d´une planorbe pour *S. Mansoni*, d´un oncomelania pour *S. japonicum*, et enfin d´une physopsis pour *S. intercalatum*.

Pendant un mois, cette minuscule larve va évoluer chez le mollusque d´abord sous forme de sporokyste dont les cellules germinales produisent des sporokystes secondaires qui migrent dans le réseau sanguin hépatopancréatique où les cellules germinales se divisent formant des cercaires. Leur libération se fait sur un rythme circadien. Leur activité est stimulée par des turbulences de l´eau, des ombres et produits chimiques présents sur la peau humaine. L´homme se contamine, non par l´absorption de l´eau polluée, mais par immersion, même très partielle, dans l´eau infestée de cercaires, au cours d´une baignade, d´une marche, les pieds nus, en terrain inondé. Les parasites traversent la peau, la cercaire se transforme en schistosomulum, les parasites cheminent dans les lymphatiques et les veines jusqu´au cœur, atteignent les poumons où elles se transforment pour migrer vers le foie et deviennent adultes en deux mois environ. Après accouplement, les femelles fécondées se séparent du mâle et vont s´établir pour des années dans leurs réseaux veineux de prédilection, vésical pour *S. haematobium*, intestinal et hépatosplénique pour les trois autres, mais cette localisation n´est pas toujours identique. Les femelles pondent d´innombrables œufs, qui finiront par tomber dans la vessie ou l´intestin de l´hôte, prêts à contaminer à nouveau les eaux douces. Certains œufs sont bloqués et ne peuvent pas être expulsés provoquant donc la schistosomiase urinaire et/ou intestinale. Des œufs qui migrent à contre-courant seront séquestrés dans différents viscères dont le foie. Ce cycle évolutif du parasite, identique pour les quatre espèces, permet de comprendre que les enfants soient particulièrement vulnérables, lors des jeux et baignades dans les ruisseaux ou rivières, ainsi que les personnes qui doivent travailler au contact de l´eau (cultivateurs, pêcheurs). Il n´y a pas de bilharziose sans un contact hydrique et la maladie ne peut prendre naissance que là où vivent les mollusques vecteurs. Le réservoir de *S. haematobium* est strictement humain alors que les autres espèces sont des zoonoses [5, 6].

Dans le cas décrit, la patiente est originaire de la région du Nord au Cameroun, une des 2 régions septentrionales où la prévalence de la schistosomiase est d´environ 80%, la troisième région septentrionale étant celle de l´Adamaoua avec une prévalence d´environ 5% [7], ceci s´expliquerait par la situation géographique, le climat de la région du Nord étant de type tropical sahélien, avec une faible hygrométrie, une sècheresse prolongée des cours d´eau saisonniers ou Mayos, dans lesquels les populations en majorité démunies effectuent la plus grande partie de leurs activités domestiques accompagnés d´une hygiène défectueuse la plupart du temps. Si les atteintes classiques sont facilement reconnaissables parce que bien décrites, le diagnostic de la localisation rare dans le système nerveux central peut être laborieux, 60 cas décrits en 20 ans [5]. Dans le cerveau, les œufs réalisent des bilharziomes intra tissulaires d´aspect tumoraux ou infiltratifs parfois volumineux, responsables d´épilepsie, de syndrome déficitaire et d´hypertension intracrânienne. La localisation dans la moelle épinière de *S. mansoni* ou de *S. haematobium* induira un risque de myélite transverse avec paraplégie flasque exactement comme chez la fillette concernée dans notre observation. L´atteinte du cône médullaire et de la moelle dorsolombaire est probablement due à l´existence d´anastomoses entre les veines du pelvis et les plexus vertébraux avalvulaires. Trois formes sont classiquement observées: myélitique transverse aiguë ou subaiguë, compressive et radiculaire [8-11]. Ici, l´origine et la résidence de la fillette en zone d´endémie de la bilharziose urinaire en Afrique [12], et particulièrement en région septentrionale du Cameroun où *S. haematobium* est largement plus répandu [13, 14] et les œufs de schistosomes avec éperons terminaux nous confortaient sur l´espèce en cause. Par ailleurs, les conditions écologiques y seraient plus propices pour le développement des hôtes intermédiaires de *Schistosoma haematobium* au détriment de *S. mansoni*.

De plus, il a été justifié que le taux d´infestation élevé chez *S. haematobium* serait dû à la dispersion facile des œufs du parasite par rapport aux œufs de *S. mansoni* dont les selles doivent au préalable subir une complète dilution avant de libérer les œufs pour être disséminés par la suite [15]. Le retard diagnostique après les premiers symptômes neurologiques est en moyenne de 1 mois, mais peut prendre jusqu´à 6 ans après l´infestation. Ce diagnostic doit être évoqué chez les patients ayant séjourné en zone d´endémie, même en l´absence de signes ou d´antécédents de bilharziose intestinale ou urinaire [16]. Le liquide céphalorachidien peut être normal ou montrer une pléiocytose avec hyperprotéinorachie. La sérologie tant sanguine que du liquide céphalorachidien n´est pas toujours spécifique et l´imagerie médicale, parfois prise en défaut en cas de myélite transverse, peut montrer des images évocatrices de granulomes bilharziens, notamment au niveau du cône médullaire qui sera élargi, tuméfié et irrégulier [9, 17].

Toutefois, le diagnostic de certitude n´est apporté que par l´histologie médullaire qui révèle l´existence d´œufs de schistosomes. Généralement, l´embryon ou miracidium déclenche la formation d´un granulome giganto-cellulaire qui évolue vers la fibrose avec destruction de la coque ovulaire et du miracidium. Plus tard, on assiste à une calcification définitive du granulome. Le granulome s´organise en trois zones concentriques avec au centre des débris ovulaires puis une couronne de macrophages. Il s´y associe des polynucléaires éosinophiles et des cellules géantes, l´ensemble est enfin entouré par une zone fibreuse externe [18, 19]. Il est à noter qu´au stade larvaire ou adulte, le parasite induit uniquement des réactions de défense de l´hôte qui aboutissent à la destruction du parasite.

Actuellement, pour la thérapeutique de toutes les formes de bilharziose, l´on dispose du Praziquantel qui est l´un des médicaments efficaces et sûrs pouvant être administrés par voie orale en prise unique de 40 mg/kg de poids corporel. Certaines atteintes, surtout urogénitales, peuvent nécessiter un traitement chirurgical [6]. La prophylaxie est très difficile. Il est recommandé d´éviter la pollution hydrique, et aussi tout contact avec une eau infestée. Ceci suppose une longue éducation et des mesures d´hygiène qui se heurtent aux habitudes ancestrales comme aux impératifs de la vie quotidienne. C´est ainsi que nous notons pour le déplorer la perte de vue du sujet de notre observation. Traiter des millions et des millions de sujets, sans cesse réinfestés, représente une immense entreprise pour les politiques de santé publique [3]. L´évolution des bilharzioses est très variée avec des interactions soit bactériennes avec les salmonelles ou les shigelles, soit virale avec le virus de l´immunodéficience humaine. L´association avec les cancers est assez documentée par divers auteurs [18, 20].

## Conclusion

Les défis de l´implémentation de la prise en charge efficace des bilharzioses restent entiers dans les pays situés en zones endémiques. Si le diagnostic des formes classiques est évident, la recherche des formes rares en général et de localisation neuroméningée en particulier devrait préoccuper les professionnels de la santé qui gagneraient à évoluer dans une approche pluridisciplinaire.
